# The conserved Phe GH5 of importance for hemoglobin intersubunit contact is mutated in gadoid fish

**DOI:** 10.1186/1471-2148-14-54

**Published:** 2014-03-21

**Authors:** Øivind Andersen, Maria Cristina De Rosa, Prakash Yadav, Davide Pirolli, Jorge MO Fernandes, Paul R Berg, Sissel Jentoft, Carl Andrè

**Affiliations:** 1 Nofima, P.O. Box 5010, Oslo N-1430 Ås, Norway; 2 Department of Animal and Aquaculture Sciences, Norwegian University of Life Sciences, P.O. Box 5003, Oslo N-1430 Ås, Norway; 3 Institute of Chemistry of Molecular Recognition - CNR and Institute of Biochemistry and Clinical Biochemistry, Catholic University of Rome, Rome 00168, Italy; 4 Institute of Biochemistry and Clinical Biochemistry, Catholic University of Rome, Rome 00168, Italy; 5 Faculty of Biosciences and Aquaculture, University of Nordland, P.O. Box 1490, Bodø 8049, Norway; 6 Centre for Ecological and Evolutionary Synthesis (CEES), Department of Biology, University of Oslo, Blindern, P.O. Box 1066, N-0316 Oslo, Norway; 7 Department of Biological and Environmental Sciences, University of Gothenburg Tjärnö, Strömstad SE-452 96, Sweden

**Keywords:** Atlantic cod, Hemoglobin, Polymorphism, Coelacanth, Spotted gar, Positive selection

## Abstract

**Background:**

Functionality of the tetrameric hemoglobin molecule seems to be determined by a few amino acids located in key positions. Oxygen binding encompasses structural changes at the interfaces between the α1β2 and α2β1 dimers, but also subunit interactions are important for the oxygen binding affinity and stability. The latter packing contacts include the conserved Arg B12 interacting with Phe GH5, which is replaced by Leu and Tyr in the α^*A*^ and α^*D*^ chains, respectively, of birds and reptiles.

**Results:**

Searching all known hemoglobins from a variety of gnathostome species (jawed vertebrates) revealed the almost invariant Arg B12 coded by the AGG triplet positioned at an exon-intron boundary. Rare substitutions of Arg B12 in the gnathostome β globins were found in pig, tree shrew and scaled reptiles. Phe GH5 is also highly conserved in the β globins, except for the Leu replacement in the β1 globin of five marine gadoid species, gilthead seabream and the Comoran coelacanth, while Cys and Ile were found in burbot and yellow croaker, respectively. Atlantic cod β1 globin showed a Leu/Met polymorphism at position GH5 dominated by the Met variant in northwest-Atlantic populations that was rarely found in northeast-Atlantic cod. Site-specific analyses identified six consensus codons under positive selection, including 122β(GH5), indicating that the amino acid changes identified at this position may offer an adaptive advantage. In fact, computational mutation analysis showed that the replacement of Phe GH5 with Leu or Cys decreased the number of van der Waals contacts essentially in the deoxy form that probably causes a slight increase in the oxygen binding affinity.

**Conclusions:**

The almost invariant Arg B12 and the AGG codon seem to be important for the packing contacts and pre-mRNA processing, respectively, but the rare mutations identified might be beneficial. The Leu122β1(GH5)Met and Met55β1(D6)Val polymorphisms in Atlantic cod hemoglobin modify the intradimer contacts B12-GH5 and H2-D6, while amino acid replacements at these positions in avian hemoglobin seem to be evolutionary adaptive in air-breathing vertebrates. The results support the theory that adaptive changes in hemoglobin functions are caused by a few substitutions at key positions.

## Background

The hemoglobin molecule has evidently been optimized for oxygen binding under vastly different environmental and physiological conditions by the structural and functional divergence of the vertebrate globin chains [1-3]. The tetrameric hemoglobin consists of two α and two β subunits each containing eight alpha helices (A-H), and the amino acids are numbered either from the N-terminus (excluding the N-terminal Met) or according to helical positions. Whereas the amino acid sequences of both α and β subunits are highly variable with very few invariant positions, adaptive modifications of hemoglobin functions seems to be attributable to a very small number of amino acid substitutions at key positions [4]. These genetically based adaptations have evolved under the influence of natural selection and involve adjustments in heme-protein contacts, intersubunit interactions and binding sites for heterotropic ligands [1,5-7]. The cooperative oxygen binding results from the allosteric equilibrium between the low-affinity T (deoxy) state and the high-affinity R (oxy) state, and the α1β2 and α2β1 dimeric interfaces undergo the principal changes during the deoxy-to-oxy transition [8-10]. In addition to these sliding contacts, the oxygen binding also involves the α1β1 and α2β2 subunit contacts, which play a key role in stabilizing the bound oxygen [11,12]. Several studies of human hemoglobin mutations have documented that even small changes in these packing contacts may affect hemoglobin stability and oxygen binding affinity [13-16]. Further, allosteric effects of chloride ions at the intradimer interfaces cause significant changes in the rates of proton exchange upon ligand binding [17]. Intriguingly, the Leu55β(D6)- > Ser and Pro119α(H2)- > Ala replacements at the α1β1 interface in the bar-headed (*Anser indicus*) and Andean geese (*Chloephaga melanoptera*), respectively, were found to increase the hemoglobin oxygen affinity in these high-altitude species by the elimination of intersubunit contacts [18-21]. Correspondingly, replacing Met with the smaller Val residue in position 55 of the polymorphic β1 globin of Atlantic cod (*Gadus morhua*) was predicted to increase the intrinsic oxygen binding affinity as demonstrated in the human Met55β- > Ser mutant [20-22].

Human hemoglobin mutants have demonstrated the importance of the interaction between Arg B12 and Phe GH5 at the α1β1 and α2β2 interfaces for proper stability and function. Replacement of Arg with the smaller Lys residue containing only two N atoms caused slight anemia in Chinese Hb Kairouan (Arg31α- > Lys) mutants [23], while normal functional properties was found in the unstable Hb Prato (Arg31α- > Ser) mutant [24]. Corresponding Arg B12 mutations in the β globin are found in the unstable human Hb Tacoma (Arg30β- > Ser), but also in the rat zero β globin [25,26], while the mutant protein and transcript were undetectable in human Hb Monroe (Arg30β- > Thr) [27]. The importance of the interacting Phe GH5 was demonstrated by the polar Ser replacement in Hb Caruaru (Phe122β- > Ser) causing chronic haemolytic anaemia [28], but the stability and oxygen binding affinity of Hb Bushey (Phe122β- > Leu) were identical to those of HbA [29]. Phe GH5 seems to be highly conserved in the β chains of other tetrapods, but has been replaced by Leu and Tyr in the αA and αD chains, respectively, of birds and reptiles (Sauropsida) [30]. Phe GH5 mutations in human α globin include Hb Foggia (Phe117α- > Ser) exhibiting a phenotype typical of α thalassemia and was found to impair interactions with both β globin and the Alpha-Hemoglobin Stabilizing Protein (AHSP) [31,32].

A Leu122(GH5)Met polymorphism was recently reported in the Atlantic cod β1 globin, which also harbors the polymorphic positions Met55(D6)Val and Lys62(E6)Ala that modify the oxygen binding properties of the HbI-1 and HbI-2 isoforms [22,33]. This marine fish is widely distributed in temperate and Arctic waters in the North Atlantic, and the HbI-1 (Met55-Lys62) and HbI-2 (Val55-Ala62) variants dominate in southern and northern populations, respectively [22,34,35]. It is plausible that the diversification of Atlantic cod globin has been driven by positive selection, since these non-synonymous mutations alter hemoglobin function and likely confer an adaptive advantage. In protein coding genes, the ratio (ω) between non-synonymous (dN) and synonymous (dS) substitution rates is related to evolutionary constraints at the protein level [37]. A value of ω > 1 indicates positive Darwinian selection, whereas ω < 1 suggests negative selection. To gain insight into the evolutionary history and the functional implications of Phe GH5 mutations, we 1) searched for amino acid replacements at this position in gnathostome β globins, 2) modeled the structural variants of Atlantic cod hemoglobin, 3) examined the distribution of the Leu122β1Met polymorphism in trans-Atlantic cod populations, and 4) investigated genetic signatures of positive selection amongst gadiform β1 sequences.

## Results

### Invariant Arg31α and novel Phe122β mutations

We searched all known hemoglobin sequences from a broad range of gnathostome species for conservation of the interacting residues Arg B12 and Phe GH5, which correspond to positions 31α/30β and 117α/122β, respectively (Additional file 1: Figure S1). Arg B12 was invariantly found in all α globins examined and was also highly conserved in the β globins with a few exceptions. Arg was replaced by Asn and Ser in the pig β-like and in tree shrew β globin, respectively, while Gly, Cys, Asn and Lys substitutions were found in the β1 and β2 globins of scaled reptiles (Squamata) (Additional file 2: Table S1).

**Figure 1:**
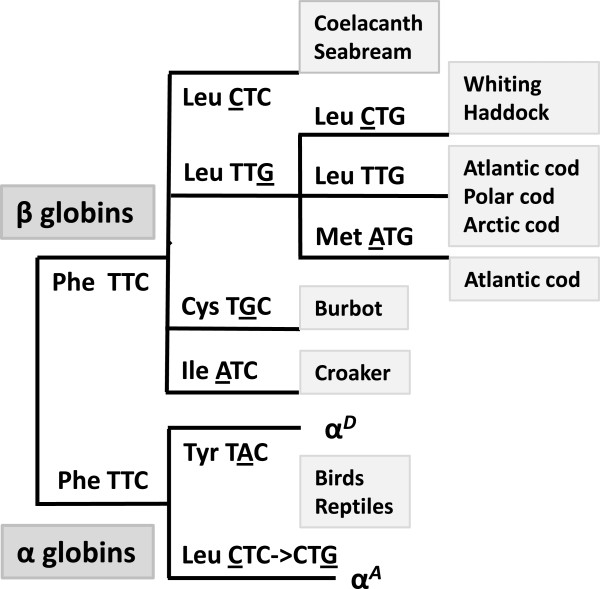
**Mutations and amino acid replacements identified at position GH5 in gnathostome α and β globins.** Possible sequential point mutations (underlined) are indicated, while ignoring any phylogenetic relationship between the given species.

Whereas Phe GH5 has been replaced by Leu or Tyr in the sauropsid α globins [30], the position has been highly conserved in the β globins with a very few exceptions (Figure 1). We identified a Phe- > Leu substitution in the β1 globin of five marine gadoid species and gilthead seabream (*Sparus aurata*), but also the lobe-finned fish Comoran coelacanth (*Latimeria chalumnae*). Further, the freshwater gadiform species burbot (*Lota lota*) β1 globin possessed Cys at position GH5, while Ile was identified in the large yellow croaker (*Larimichthys crocea*). Gadoids possess up to five different β globins [22,33,38], but replacements of Phe GH5 were only found in the β1 chain. Similarly, the second β globin of gilthead seabream and Comoran coelacanth contains the conserved Phe GH5. Based on the nucleotide sequences available we predicted the sequential point mutations causing the identified Phe GH5 replacements (Figure 1).

### Modelled 3D structure of mutated α1β1 contacts

**Figure 2:**
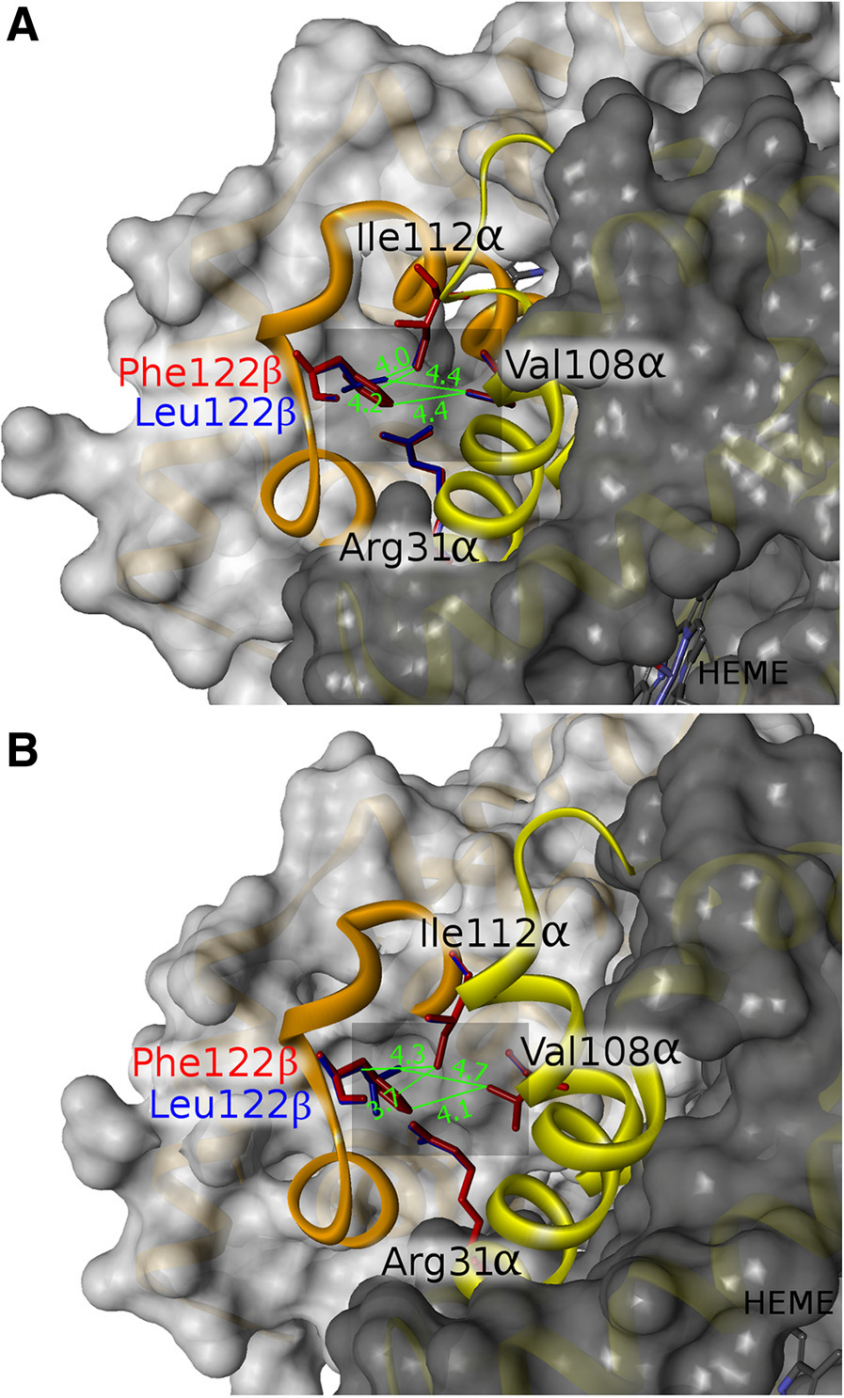
**Modeling of Phe122β1- > Leu replacement in Atlantic cod hemoglobin.** Superimposition of the three-dimensional model structures of Phe122β (red) and Leu122β (blue) variants in **A)** deoxy- and **B)** oxy-states. Surface structure of the α1β1 dimer is displayed with the α- and β-chains highlighted in dark gray and light gray, respectively. The B, G helices of α-chain (yellow) and the G, H helices and GH corner of β-chain (orange) are shown in ribbon representation. The closest distances from 122β1 residue to the α-chain are shown in Å.

Computational mutation analysis was performed to examine the structural changes at the α1β1 interface in the Atlantic cod hemoglobin caused by replacing Phe122β (GH5) with Leu or Met, but also with the Cys residue identified in burbot. The involvement of the amino acid replacement in the allosteric transition was addressed by generating computational models of both the T- and R-states of the Atlantic cod Hb3 tetramer comprising α1-α1-β1-β1 [38]. The resulting structures passed all stereochemical and geometric checks in PROCHECK and showed that Phe122β forms van der Waals contacts across the interface with Arg31α, Val108α and Ile112α in both T- and R- states (Figure 2, Table 1). Both the Phe- > Leu and Phe- > Cys substitutions slightly decreased the number of intersubunit contacts in the T-state compared to the R-state, while the Phe- > Met change did not promote any difference in contacts at the α1β1 interface. We therefore predict a mild destabilization of the T-state as a consequence of the Leu or Cys replacement that might slightly increase the oxygen binding affinity of these variants. In contrast, replacing Phe122β with Leu in human HbA caused no difference in the number of van der Waals contacts with Arg31α and Val107α in the T-and R-state (Table 1). Consistently, the Hb-Bushey mutant exhibited identical stability and oxygen binding affinity as HbA [29].

**Table 1:** Residues of α chain and number of atoms at a maximum distance of 4.5 Å from β GH5 position in Atlantic cod, burbot and human hemoglobin at the deoxy (T) and oxy (R) state

		**Contacts**	**Number of atoms**	
			**Deoxy**	**Oxy**
**Atlantic cod**	**Phe**	Arg31α	3	2
		Val108α	1	1
		Ile112 α	1	1
	**Leu**	Arg31α	3	2
		Val108α	-	1
		Ile112 α	1	1
	**Met**	Arg31α	3	2
		Val108α	1	1
		Ile112 α	1	1
**Burbot**	**Cys**	Arg31α	3	3
**Human**	**Phe**	Arg31α	3	3
		Val107α	1	1
	**Leu**	Arg31α	3	3
		Val107α	1	1
	**Ser**	Arg31α	3	3

### Leu122β1Met polymorphism in trans-Atlantic cod populations

**Figure 3:**
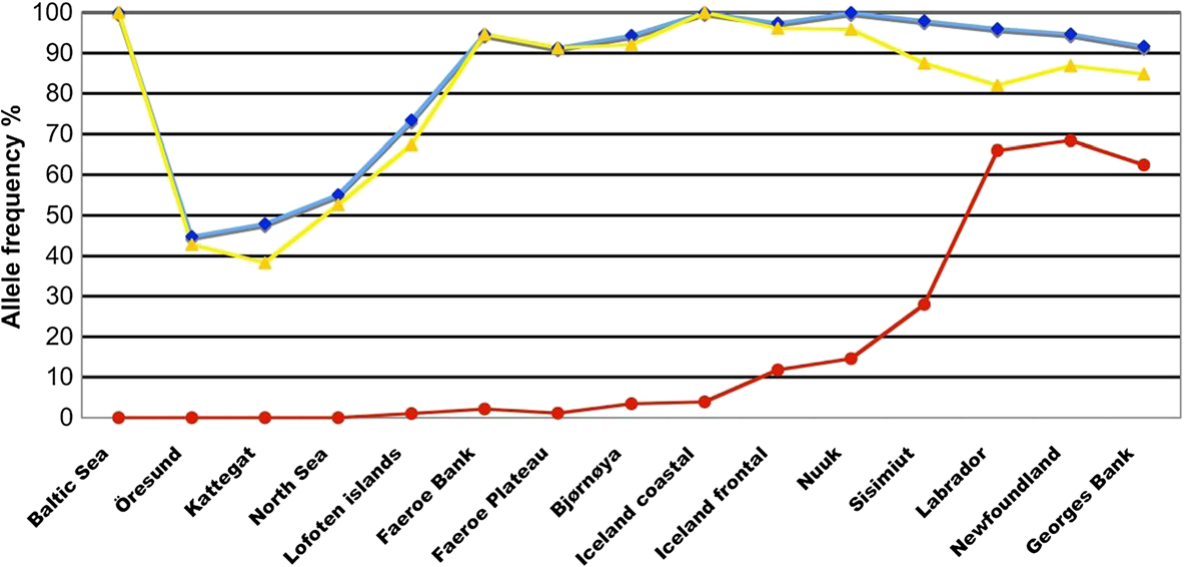
**Allele frequencies of the three polymorphic sites in Atlantic cod β1 globin from samples collected across the North Atlantic.** Met55Val (Val: blue line), Lys62Ala (Ala: yellow line) and Leu122Met (Met: red line). See Additional file 3: Table S2 for sampling locations and Additional file 5: Table S4 for SNP alleles frequencies.

The distribution of the Leu122β1(GH5)Met polymorphism in trans-Atlantic populations of Atlantic cod was examined by SNP genotyping a total of 560 adult fish representing 15 populations (Additional file 3: Table S2). Population pairwise *F*_ST_ values demonstrated a clear genetic separation between northeastern and northwestern cod samples with intermediate Icelandic and Greenland populations (Additional file 4: Table S3). The Met allele dominated in the three Canadian populations (66.0-68.4%), and almost 50% of the Labrador and Newfoundland cod were homozygous for this allele (Figure 3). The allele frequency of Met122 decreased to 28.0 and 14.5%, respectively, in the Sisimiut and Nuuk populations, and only one Greenland fish was identified as homozygous Met. Even lower frequencies of this allele were found in Icelandic coastal and frontal populations (7.8%), Northeast Arctic cod (NEAC) population in Barents Sea (3%), Faroe bank and plateau populations (1.5%) and in spawning NEAC fish outside Lofoten islands (1%), while the Met allele was not identified in the North Sea, Kattegat and Baltic Sea populations (Additional file 5: Table S4). All samples conformed to Hardy-Weinberg expectations (Additional file 6: Table S5).

The polymorphic positions Met55Val and Lys62Ala of Atlantic cod β1 globin that discriminate between the HbI-1 (Met55-Lys62) and HbI-2 (Val55-Ala62) isoforms [22] were included in the genotyping to examine the distribution of the haplotypes. The Met122 variant was not identified in any HbI-1 fish and so exhibited the single haplotype Met55-Lys62-Leu122, while the Leu122Met polymorphism in the HbI-2 fish resulted in the two haplotypes Val55-Ala62-Leu122 and Val55-Ala62–Met122. The latter haplotype was rarely found in northeast-Atlantic populations, while the Val55-Lys62-Met122 recombination was identified in two individuals from Labrador and Georges Bay, and a Sisimiut recombinant exhibited the Val55-Lys62-Leu122 haplotype.

### Positive selection

To detect genetic signatures of positive selection acting on the evolution of the β1 globin gene in Gadiformes, a site-specific likelihood analysis was performed using PAML and Datamonkey (Table 2). The one-ratio M0 model, which assumes the same ω ratio between non-synonymous (dN) and synonymous (dS) substitutions for all branches, had a log-likelihood value of −1092.88 with ω = 0.26. The log-likelihood value for the M3 model with three discrete site classes was −1064.41 with ω_0_ = 0.08, ω_1_ = 0.08 and ω_2_ = 2.21. Comparison of the two models using a likelihood ratio test revealed that model M3 provided a significantly better fit to our data set. When compared to a χ^2^ distribution with 4 degrees of freedom (df), the difference in log-likelihood values (2ΔLnL) of 56.94 supports the rejection of the one-ratio model M0 (p = 0). Similarly, the other two evolution models that allowed for positive selection fitted the data better than those that did not (M2a versus M1a, p = 0.05; M8 versus M7, p = 0.01). Models M2a, and M3 identified positively selected sites at positions 6 (p = 0.995), 9 (p = 0.999), 13 (p = 0.997), 23 (p = 0.995), 55 (p = 0.995), 62 (p = 1), 122 (p = 1) and 123 (p = 0.993) with a ω ratio of 2.22 and 2.21, respectively. The same codons were identified in model M8 with ω = 2.22 and similar probability values greater than 0.99. The REL algorithm implemented in Datamonkey recognized positively selected sites with a Bayes factor greater than 50 at positions 6, 9, 13, 55, 62 and 122. The consensus from all methods indicated that codons 6 (Ala, Thr or Tyr), 9 (Arg, Ser, Thr or Lys), 13 (Ala, Thr or Gln), 55 (Met, Val or Leu), 62 (Lys, Ala, Asn, Gln or Arg) and 122 (Leu, Met or Cys) are under positive selection (Table 2; Additional file 7: Figure S2).

**Table 2:** Identification of positively selected sites in gadoids and burbot β1 globin by maximum likelihood analysis using various models of evolution

**Method**	**Model**	**Parameter estimates**	**Ln likelihood**	**Model comparison**	**Positively selected sites**^**a**^
CODEML	M0: neutral	ω = 0.26	−1092.88		None
	M1a: nearly neutral	ω_0_ = 0.05, ω_1_ = 1.00	−1067.42		Not allowed
		p_0_ = 0.80, p_1_ = 0.20			
	M2a: positive selection	ω_0_ = 0.08, ω_1_ = 1.00, ω_2_ = 2.22	−1064.41	M2 vs M1	6, 9, 13, 23, 55, 62, 122, 123
				2ΔLnL = 6.02, df = 2, p = 0.05	
		p_0_ = 0.86, p_1_ = 0, p_2_ = 0.14			
	M3: discrete	ω_0_ = 0.08, ω_1_ = 0.08, ω_2_ = 2.21	−1064.41	M3 vs M0	6, 9, 13, 23, 55, 62, 122, 123
		p_0_ = 0.40, p_1_ = 0.46, p_2_ = 0.14		2ΔLnL = 56.94, df = 4, p = 0	
	M7: β	p = 0.07, q = 0.21	−1069.19		Not allowed
	M8: β + ωS > 1	p = 9.24, q = 99	−1064.45	M8 vs M7	6, 9, 13, 23, 55, 62, 122, 123
				2ΔLnL = 9.48, df = 2, p = 0.01	
		ω = 2.22			
		p_0_ = 0.86, p_1_ = 0.14			
HYPHY	REL	ω = 2.72			6, 9, 13, 55, 62, 122

^a^Only positively selected codons with Bayesian posterior probabilities equal or greater than 99% are indicated.

## Discussion

The vast phylogenetic variation and adaptive modifications of the hemoglobin molecule have fascinated scientists since the pioneering work of Braunitzer [39] identifying only eight invariant positions in multiple vertebrate hemoglobins. The post-genomic era has later provided numerous hemoglobin protein and gene sequences from a variety of organisms and even from extinct species to investigate evolutionary conserved positions as well as adaptive changes in specific lineages. Here we show that all α globins available from the gnathostomes exhibit the invariant Arg B12, which is predominantly coded by the AGG codon, whereas the six different Arg codons are found in other positions of the α globins in warm- and cold-blooded vertebrates [40]. Genomic sequences revealed that the Arg B12 codon spans the exon 1-exon 2 boundary of a phase 2 intron, and AG↓G is the most frequent signal for exon splicing [41]. Hence, the functional constraints of the invariant position are accompanied by independent requirements of exon splicing. Accordingly, the AGG- > ACG mutation in human Hb Monroe (Arg30β- > Thr) inhibited pre-mRNA splicing and no mutant protein and transcript was detected [27,42]. Further, the rare AGA codon is found in the β-like δ globin gene of primates, and higher primates produce only a small amount of Hb A_2_ (α_2_δ_2_, <6% of total hemoglobin), while *δ globin* is a silent gene in Old World monkeys [43,44]. This contrasts with the gadoid *β1 globin*, which also has the AGA codon, but is highly expressed in the adult Atlantic cod at similar levels of β2 globin containing the conserved AGG codon [38,45]. The additional mutations identified at this position in β globins of pig, tree shrew and scaled reptiles raise the question about the importance of this codon for correct mRNA splicing. Possible effects of these amino acid replacements on hemoglobin function warrant further studies although the multiple changes identified at subunit contacts and heme contacts in cobra and sea snake hemoglobin appeared compatible with conserved overall functional properties [46,47]. The lizard β1 and β2 globins are probably products of a lizard-specific duplication event, and the phylogenetic positions of the β paralogs suggest that the common reptile ancestor may have possessed a fairly diverse repertoire of β-like globin genes [48].

The highly conserved Arg B12 interacts with Phe GH5, which has been replaced by Leu and Tyr, respectively, in the sauropsid α^*A*^ and α^*D*^ chains forming the major HbA and minor HbD together with a common β chain [30,49]. The higher intrinsic oxygen affinity of avian HbD compared to HbA was proposed to involve substitutions at three positions in the α1β1 packing contacts, including the Leu117α(GH5)- > Tyr change [30]. This mutation might represent an evolutionary adaptation to air breathing in reptiles and birds, while the reported Leu55β- > Ser and Pro119α- > Ala replacements have further increased the oxygen affinity in high-altitude birds [18,19]. We found Phe GH5 in the β globins of all sarcopterygians examined, except for the Phe- > Leu replacement in a “living fossil”; the Comoran coelacanth. It should be noted that the Phe122β- > Leu replacement is also found in the human Hb Bushey mutant, but the stability and binding affinity were shown to be identical to normal HbA [29]. On the other hand, multiple heme contacts and positions involved in subunit interface contacts have been replaced in the coelacanth hemoglobin, including the loss of an α1β2 contact that might be responsible for the easy dissociation of the tetrameric molecule [50]. The coelacanth genome harbors two α and two β globin genes, and the phylogenetic tree grouped the adult β1 and embryonic β2 globins together with amphibian embryonic β-chains in a clade that was lost in the amniote tetrapods [51,52]. Whereas the Phe122β- > Leu mutation has disappeared in the amniote β globins, the same amino acid change has occurred in the sauropsid α^*A*^ chain. Our site-specific analyses identified six consensus codons under positive selection in gadiform β1 globins, and several amino acid substitutions observed at these positively selected sites produce significant changes in charge, size or hydrophobicity, which may affect hemoglobin function. Intriguingly, the identified sites under positive selection include positions 55, 62 and 122, which are polymorphic in Atlantic cod; amino acid substitutions at two of these positions in avian hemoglobin seem to offer a selective advantage in air-breathing vertebrates. Altogether, the results support the theory of Perutz [4] that adaptive changes in hemoglobin functions are caused by a few amino acid substitutions at key positions.

The adaptability of Atlantic cod to variable environmental conditions in Arctic and temperate North Atlantic waters seems to involve several genomic regions containing multiple polymorphic genes [22,53-56]. The selective advantage of possessing functionally different hemoglobin isoforms has been well documented in Atlantic cod, although contradicting results exist [22,57-61]. The HbI-1 and HbI-2 allelic variants differ in oxygen binding affinity and temperature sensitivity and are differentially distributed along a temperature gradient in northeast Atlantic populations [34-36]. The HbI-2 variant predominates in northwest-Atlantic waters, but the Met122β1Leu polymorphism in these populations might further increase the plasticity of this successful species to fluctuating environments. Paleoecological modelling and genetic studies of nuclear and mitochondrial markers suggest that cod populations have survived as least for 100, 000 years on both sides of the Atlantic [62-65]. The Leu122β1Met polymorphism likely originated in the Canadian populations and has expanded in these waters probably over a short historical time and driven by positive selection acting on this codon. Consistently, strong temporal shifts were observed in several gene-associated SNP loci in Canadian populations over an 80-year period indicating ongoing selection over short time-scales [66]. The intermediate frequencies of the Met122 allele in West Greenland populations and the rare distribution in Icelandic populations are supported by the occasional migration of adult cod from Canada to West-Greenland waters and the age-specific migration towards East Greenland and Iceland [67,68]. Consistently, postglacial gene flow was suggested by the spatiotemporal SNP analysis of trans-Atlantic cod populations demonstrating that samples from West Greenland offshore showed the greatest genetic affinity to Canada [69].

## Conclusions

The importance of the α1β1 and α2β2 subunit contacts for hemoglobin stability and oxygen binding affinity is strongly supported by the conservation of the interacting residues Arg B12 and Phe GH5 in the gnathostomes. On the other hand, amino acid replacements identified at these positions in β globins of scaled reptiles and gadiform fishes might offer a selective advantage under certain conditions as indicated by the modeled interactions and genetic signatures of positive selection in the latter group. Intriguingly, the intradimer contacts B12-GH5 and H2-D6 are both polymorphic in Atlantic cod, while amino acid replacements at these positions in avian hemoglobin seem to be beneficial for air-breathing.

## Methods

### Vertebrate globin sequences

Conserved positions in the α and β globins available from gnathostome vertebrates were analysed by multiple sequence alignment of sequences available at http://www.ncbi.nlm.nih.gov and http://Ensembl.org using the BLAST tool on NCBI. Globin transcripts from whiting (*Merlangius merlangus*), haddock (*Melanogrammus aeglefinus*) and burbot were retrieved from the cod genome database at http://codgenome.no.

### Protein modeling

Sequences of globin chains with known 3D structure were selected based upon similarity with Atlantic cod α1 and β1 globins using PSI-BLAST (blast.ncbi.nlm.nih.gov/Blast). The homology model of T-state Atlantic cod hemoglobin was based on the structures of Antarctic rock cod (*Trematomus bernacchii*) (Protein Data Bank (PDB) 1HBH), the Dusky notothen (*Trematomus newnesi*) (PDB 2AA1), bluefin tuna (*Thunnus thynnus*) (PDB 1V4W), and rainbow trout (*Oncorhynchus mykiss*) (PDB 1OUT). For R-state Atlantic cod hemoglobin, the structures of the red-tailed Brycon (*Brycon cephalus*) (PDB 3BCQ), bluefin tuna (PDB 1V4U) and rainbow trout (PDB 1OUU) were used as templates. Sequence alignments were carried out using ClustalW (clustalw.genome.ad.jp/). Based on ClustalW alignments, three-dimensional models were generated by comparative protein modeling with MODELLER program [70] as implemented in Discovery Studio 3.5 (Accelrys Inc.). Twenty models, optimized by a short simulated annealing refinement protocol available in MODELLER, were generated for each globin chain. The geometrical consistency of the model was evaluated based on PDF violations provided by the program. The BUILD_MUTANT module of MODELLER was then used for computational mutagenesis experiments. One hundred alternative conformations of each hemoglobin mutant at position 122β position were generated by the program. After examination of the models with Discovery Studio (Accelrys Inc.), a representative model from each set was chosen that had few restraint violations and favourable stereochemical properties as determined using PROCHECK [71].

### Population genotyping and analyses

A total of 560 adult Atlantic cod were collected during 2001–2011 from 15 trans-Atlantic populations (Additional file 2: Table S1). Genomic DNA was extracted using the DNeasy blood and tissue kit (Qiagen) from fin clips, spleen or muscle tissues stored in 95% ethanol or RNAlater (Qiagen). The β1 globin variants were genotyped by single nucleotide polymorphism (SNP) analysis. MassArrayTyper (Version 4 running Assay Editor 4.0.20.5) was used to design primers for multiplex PCR to amplify a region containing Met55Val and Lys62Ala, and a region containing the Met122Leu polymorphism. The sequences for the two primer pairs are: Sense, 5′-acgttggatgtttggcgacctgagcaccga-3′, antisense, 5′-acgttggatgtggtccagagccgtcctca-3′, and sense; 5′- acgttggatgtttcagctgctgtgtgagtg-3′, antisense; 5′-acgttggatgacaggtacttctgccacgc-3′. The SNPs were determined using downstream or upstream extension primers with the respective sequences: 5′- accgacgccgctatt-3′, 5′-ggccacgacgccgtgc-3′ and 5′-ctgcatctccgggctca-3′. Samples were genotyped using a MassArray4 instrument, and the genotypes were assigned with MassArrayTyper (Version 4 running Typer Analyzer 4.0.22.67) and manually inspected using the MassArray Typer v. 3.3 software. SNP heterozygosities, linkage disequilibrium and population differentiation (*F*_ST_), as well as statistical significances were calculated using Genepop 4.0.10 [72].

### Analyses of positive selection

A 146-codon alignment spanning the full-length coding sequence of Atlantic cod β1 globin was obtained with MUSCLE (http://www.ebi.ac.uk/Tools/msa/muscle/) using a total of 13 sequences from six gadiformes, namely Atlantic cod, whiting, haddock, burbot, polar cod (*Boreogadus saida*) and Arctic cod (*Arctogadus glacialis*). This alignment was used for Bayesian inference of phylogeny, as previously reported [73]. The GTR evolutionary model with gamma-distributed rate variation across sites and a proportion of invariable sites was selected and 4 Markov chains were run for 500,000 generations sampling every 10^th^ generation. A consensus tree was built after burning the first 5,000 trees. Individual sites under positive (diversifying) selection were identified using the maximum likelihood methods implemented in the CODEML program of PAML v4.7 [74], as detailed in [75]. We investigated the ratio (ω) between non-synonymous (dN) and synonymous (dS) substitutions using several branch-site models, which allow ω to vary among codons in the β1 globin protein. The models of positive selection used were M2a with three site classes (ω =1, 0 < ω < 1 and ω > 1), M3 with three discrete site classes of different ω values and M8 with a β distribution of sites, including one class site with ω > 1. These were then compared with the appropriate nested neutral or nearly neutral evolution models M0, M1a and M7 by likelihood ratio tests. Model M0 assumes the same ω ratio for all branches in the phylogeny and for all codons in the β1 globin gene, whereas model M1a allows for two site classes (ω = 1 and 0 < ω < 1) and model M7 uses a β distribution of class sites that does not allow for selection (0 < ω < 1). Both naïve and Bayes empirical Bayes were used to determine Bayesian posterior probabilities (p) of positively selected sites. In addition, our data set was analyzed with the random effects likelihood model of molecular evolution (REL) implemented in Datamonkey to identify specific sites under positive selection [76].

## Competing interests

The authors declare that they have no competing interests.

## Authors’ contributions

ØA conceived and designed the study, and wrote the manuscript. MCDR and DP performed the computer modeling study and the generation of 3D structures. CA, PY and PRB carried out the population genetic analyses. JMOF performed the analyses of positive selection. SJ was responsible the gadoid globin sequences. All authors critically read the manuscript drafts and approved the final version of the manuscript.

## Supplementary Material

Additional file 1: Figure S1 Sequence alignment of human and Atlantic cod α and β globins. The A-H helices are indicated together with positions B12 and GH5 (arrow). The polymorphic sites Met55β1Val, Lys62β1Ala and Leu122β1Met in Atlantic cod are included. GeneBank accession numbers: Human: α; ABD95911, β; AAA16334. Atlantic cod: α1; ACV69830, β1; ACV69840. Click here for file

Additional file 2: Table S1 Arg31β(B12) replacements identified in gnathostome species. Accession number is indicated for the protein, or mRNA when available. Click here for file

Additional file 3: Table S2 Atlantic cod sample location and size. The Baltic cod were pooled into one sample in the analyses. The data storage tagged (DST) Icelandic cod have been previously described [77,78]. Click here for file

Additional file 4: Table S3 Genetic hemoglobin differentiation at three SNP loci among pairs of samples of Atlantic cod. A) Met55Val, B) Lys62Ala, C) Leu122Met. Below diagonal are pair-wise FST-values, and above diagonal statistical significance values. P-values < 0.05 are in light yellow, and P-values significant after correction for multiple testing (a’ = 0.0005) are in dark yellow. Click here for file

Additional file 5: Table S4 Allele (A) and genotype (B) frequencies of three polymorphic positions sites of Atlantic cod β1 globin in 15 trans-Atlantic populations. Click here for file

Additional file 6: Table S5 Observed and expected heterozygosity at three polymorphic positions of Atlantic cod β1 globin in 15 trans-Atlantic populations. All samples conformed to Hardy-Weinberg expectations. Click here for file

Additional file 7: Figure S2 Multiple sequence alignment of gadiform β1 globins. Nucleotides identical to *G. morhua* isoform 1 are represented by a dot. Positively selected sites with a Bayesian posterior probability greater than 0.99 are highlighted in gray. Codons 6, 9, 13, 55, 62 and 122 were identified by all evolution models tested. Predicted amino acid sequence of *G. morhua* isoform 1 together with amino acid substitutions in the positively selected codons are shown in italics above the nucleotide sequence. Genbank accession numbers for the β1 globin nucleotide sequences are as follows: *G. morhua* (FJ392683, FJ66675, FJ666977, FJ666972, FJ666976, FJ666973), *M. merlangus* (X98349), *B. saida* (HO076351, HO075251, HO075774), *A. glacialis* (DQ125476), *M. aeglefinus* (F4B3CVS02GS884) and *L. lota* (F4B3CVS02HILH5). Click here for file
