# Supplementary material for: The conserved Phe GH5 of importance for hemoglobin intersubunit contact is mutated in gadoid fish
Source: BMC Evol Biol. 2014 Mar 21;14:54. doi: 10.1186/1471-2148-14-54 (PMC3998052; doi:10.1186/1471-2148-14-54)
Supplement: Additional file 1: Figure S1 — Sequence alignment of human and Atlantic cod α and β globins. The A-H helices are indicated together with positions B12 and GH5 (arrow). The polymorphic sites Met55β1Val, Lys62β1Ala and Leu122β1Met in Atlantic cod are included. GeneBank accession numbers: Human: α; ABD95911, β; AAA16334. Atlantic cod: α1; ACV69830, β1; ACV69840. [file 1471-2148-14-54-S1.pptx]

## Slide 1
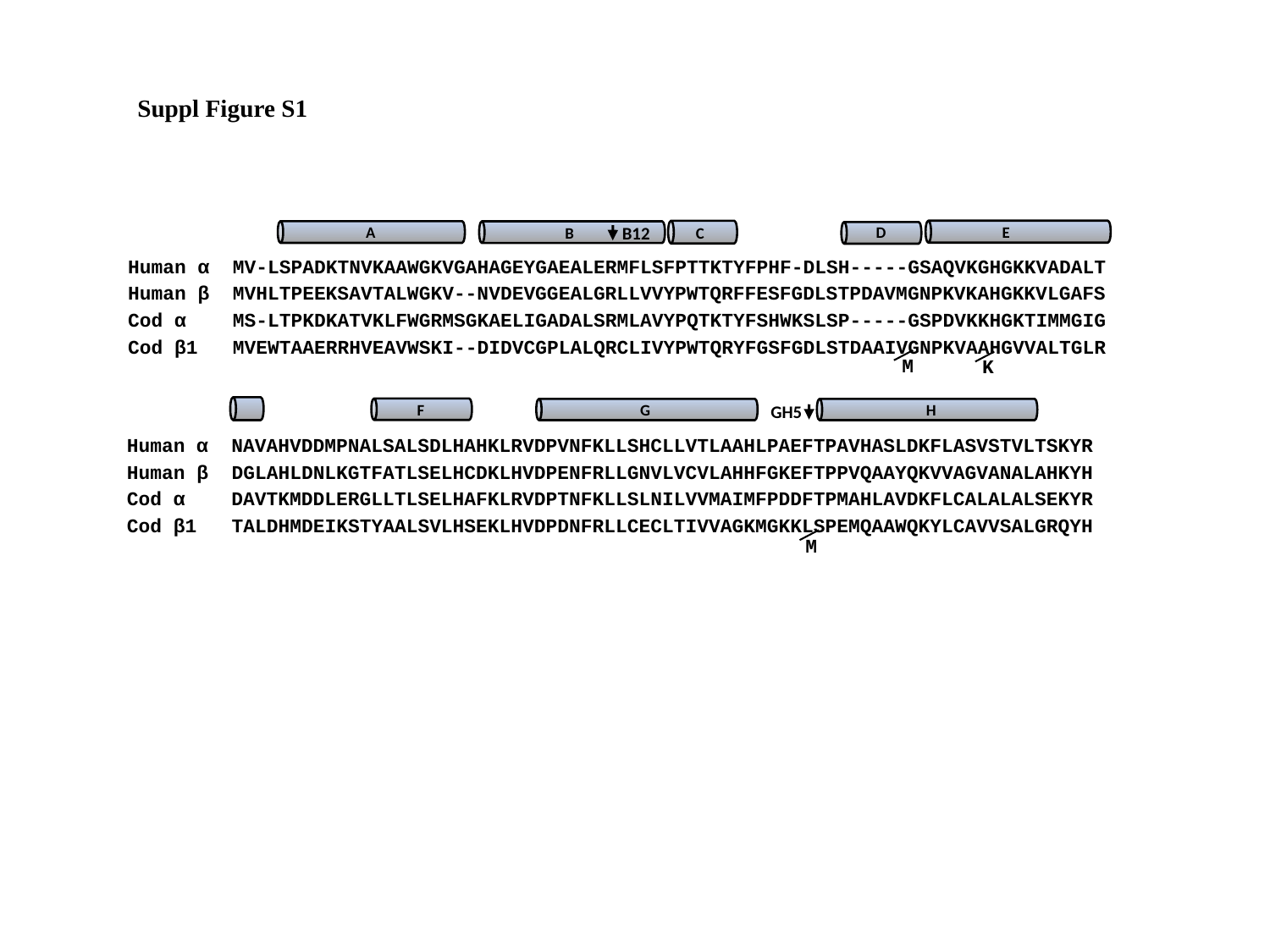

Suppl Figure S1
A
E
B12
B
D
C
M
K
Human α MV-LSPADKTNVKAAWGKVGAHAGEYGAEALERMFLSFPTTKTYFPHF-DLSH-----GSAQVKGHGKKVADALT
Human β MVHLTPEEKSAVTALWGKV--NVDEVGGEALGRLLVVYPWTQRFFESFGDLSTPDAVMGNPKVKAHGKKVLGAFS
Cod α MS-LTPKDKATVKLFWGRMSGKAELIGADALSRMLAVYPQTKTYFSHWKSLSP-----GSPDVKKHGKTIMMGIG
Cod β1 MVEWTAAERRHVEAVWSKI--DIDVCGPLALQRCLIVYPWTQRYFGSFGDLSTDAAIVGNPKVAAHGVVALTGLR
F
G
H
GH5
Human α NAVAHVDDMPNALSALSDLHAHKLRVDPVNFKLLSHCLLVTLAAHLPAEFTPAVHASLDKFLASVSTVLTSKYR
Human β DGLAHLDNLKGTFATLSELHCDKLHVDPENFRLLGNVLVCVLAHHFGKEFTPPVQAAYQKVVAGVANALAHKYH
Cod α DAVTKMDDLERGLLTLSELHAFKLRVDPTNFKLLSLNILVVMAIMFPDDFTPMAHLAVDKFLCALALALSEKYR
Cod β1 TALDHMDEIKSTYAALSVLHSEKLHVDPDNFRLLCECLTIVVAGKMGKKLSPEMQAAWQKYLCAVVSALGRQYH
M
