# Supplementary material for: The conserved Phe GH5 of importance for hemoglobin intersubunit contact is mutated in gadoid fish
Source: BMC Evol Biol. 2014 Mar 21;14:54. doi: 10.1186/1471-2148-14-54 (PMC3998052; doi:10.1186/1471-2148-14-54)
Supplement: Additional file 2: Table S1 — Arg31β(B12) replacements identified in gnathostome species. Accession number is indicated for the protein, or mRNA when available. [file 1471-2148-14-54-S2.docx]

**Supplementary table S1.**

| **Species** | **B12 residue** | **Codon** | **Accession number** |
| --- | --- | --- | --- |
| Monitor lizard β | Gly |  | P18993 |
| Common iguania β1 | Gly |  | P18987 |
| Common iguania β2 | Cys |  | P86390 |
| Green anole β | Cys | tgc | XM_003229614.1 |
| Sea snake β | Lys |  | P41332 |
| Cobra snake β2 | Lys |  | P22743 |
| Spiny-tailed lizard β1 | Asn |  | P18991 |
| Pig β-like | Lys | aag | XR_297530.1 |
| Tree shrew β | Ser | agt | XM_006151709.1 |
