# Supplementary material for: The conserved Phe GH5 of importance for hemoglobin intersubunit contact is mutated in gadoid fish
Source: BMC Evol Biol. 2014 Mar 21;14:54. doi: 10.1186/1471-2148-14-54 (PMC3998052; doi:10.1186/1471-2148-14-54)
Supplement: Additional file 3: Table S2 — Atlantic cod sample location and size. The Baltic cod were pooled into one sample in the analyses. The data storage tagged (DST) Icelandic cod have been previously described [77,78]. [file 1471-2148-14-54-S3.doc]

**Supplementary table S2**

| **Locality** | **Lat** | **Long** | **Sampling year** | **Sample size** |
| --- | --- | --- | --- | --- |
|  |  |  |  |  |
| Baltic Sea Öland | 57.40 | 17.00 | 2008 | 20 |
| Baltic Sea Gotland | 57.15 | 18.78 | 2008 | 20 |
| Baltic Sea Bornholm | 55.59 | 16.30 | 2004 | 8 |
| Öresund | 55.95 | 12.70 | 2003 | 48 |
| Kattegat | 56.90 | 12.15 | 2004 | 48 |
| North Sea | 49.9-52.3 | 0.4-3.6 | 2009 | 39 |
| Lofoten Islands | 68.18 | 13.46 | 2011 | 47 |
| Faeroe bank | 61.10 | -08.30 | 2008 | 46 |
| Faeroe plateau | 61.96 | -06.02 | 2008 | 46 |
| Bjørnøya island | 74.00 | 17.41 | 2001 | 44 |
| Iceland coastal | * | * | 2003 | 38 |
| Iceland frontal | * | * | 2003 | 38 |
| Greenland Nuuk | 64.73 | -50.45 | 2003 | 24 |
| Greenland Sisimiut | 66.84 | -52.89 | 2003 | 25 |
| Labrador | 45.74 | -58.41 | 2003 | 25 |
| Newfoundland | 52.06 | -53.39 | 2004 | 19 |
| Georges bank | 42.15 | -67.01 | 2003 | 24 |
