# Supplementary material for: The conserved Phe GH5 of importance for hemoglobin intersubunit contact is mutated in gadoid fish
Source: BMC Evol Biol. 2014 Mar 21;14:54. doi: 10.1186/1471-2148-14-54 (PMC3998052; doi:10.1186/1471-2148-14-54)
Supplement: Additional file 5: Table S4 — Allele (A) and genotype (B) frequencies of three polymorphic positions sites of Atlantic cod β1 globin in 15 trans-Atlantic populations. [file 1471-2148-14-54-S5.doc]

**Supplementary table S5**.

(A)

| **Sampling** | **Met55Val** | | **Lys62Ala** | | **Leu122Met** | |
| --- | --- | --- | --- | --- | --- | --- |
| **locations** | **G** | **A** | **C** | **A** | **T** | **A** |
|  |  |  |  |  |  |  |
| Baltic Sea | 1.00 | 0.00 | 1.00 | 0.00 | 1.00 | 0.00 |
| Öresund | 0.45 | 0.55 | 0.43 | 0.57 | 1.00 | 0.00 |
| Kattegat | 0.48 | 0.52 | 0.38 | 0.62 | 1.00 | 0.00 |
| North Sea | 0.55 | 0.45 | 0.53 | 0.47 | 1.00 | 0.00 |
| Lofoten Islands | 0.73 | 0.27 | 0.67 | 0.33 | 0.99 | 0.01 |
| Faeroe Bank | 0.95 | 0.05 | 0.95 | 0.05 | 0.98 | 0.02 |
| Faeroe Plateau | 0.91 | 0.09 | 0.91 | 0.09 | 0.99 | 0.01 |
| Bjørnøya | 0.94 | 0.06 | 0.92 | 0.08 | 0.97 | 0.03 |
| Iceland Coastal | 1.00 | 0.00 | 1.00 | 0.00 | 0.96 | 0.04 |
| Iceland Frontal | 0.97 | 0.03 | 0.96 | 0.04 | 0.88 | 0.12 |
| Nuuk | 1.00 | 0.00 | 0.96 | 0.04 | 0.85 | 0.15 |
| Sisimiut | 0.98 | 0.02 | 0.88 | 0.13 | 0.72 | 0.28 |
| Labrador | 0.96 | 0.04 | 0.82 | 0.18 | 0.34 | 0.66 |
| Newfoundland | 0.95 | 0.05 | 0.87 | 0.13 | 0.32 | 0.68 |
| Georges Bank | 0.92 | 0.08 | 0.85 | 0.15 | 0.38 | 0.63 |

(B)

| **Sampling** |  | **Met55Val** |  |  | **Lys62Ala** |  |  | **Leu122Met** |  |
| --- | --- | --- | --- | --- | --- | --- | --- | --- | --- |
| **locations** | **GG** | **GA** | **AA** | **CC** | **CA** | **AA** | **TT** | **TA** | **AA** |
|  |  |  |  |  |  |  |  |  |  |
| Baltic Sea | 1.00 | 0.00 | 0.00 | 1.00 | 0.00 | 0.00 | 1.00 | 0.00 | 0.00 |
| Öresund | 0.15 | 0.60 | 0.25 | 0.13 | 0.60 | 0.27 | 1.00 | 0.00 | 0.00 |
| Kattegat | 0.27 | 0.42 | 0.31 | 0.17 | 0.43 | 0.40 | 1.00 | 0.00 | 0.00 |
| North Sea | 0.28 | 0.54 | 0.18 | 0.23 | 0.59 | 0.18 | 1.00 | 0.00 | 0.00 |
| Lofoten Islands | 0.55 | 0.36 | 0.09 | 0.50 | 0.35 | 0.15 | 0.98 | 0.02 | 0.00 |
| Faeroe Bank | 0.89 | 0.11 | 0.00 | 0.89 | 0.11 | 0.00 | 0.96 | 0.04 | 0.00 |
| Faeroe Plateau | 0.83 | 0.17 | 0.00 | 0.83 | 0.17 | 0.00 | 0.98 | 0.02 | 0.00 |
| Bjørnøya | 0.89 | 0.11 | 0.00 | 0.86 | 0.11 | 0.02 | 0.93 | 0.07 | 0.00 |
| Iceland Coastal | 1.00 | 0.00 | 0.00 | 1.00 | 0.00 | 0.00 | 0.92 | 0.08 | 0.00 |
| Iceland Frontal | 0.95 | 0.05 | 0.00 | 0.92 | 0.08 | 0.00 | 0.76 | 0.24 | 0.00 |
| Nuuk | 1.00 | 0.00 | 0.00 | 0.92 | 0.08 | 0.00 | 0.75 | 0.21 | 0.04 |
| Sisimiut | 0.96 | 0.04 | 0.00 | 0.79 | 0.17 | 0.04 | 0.44 | 0.56 | 0.00 |
| Labrador | 0.92 | 0.08 | 0.00 | 0.64 | 0.36 | 0.00 | 0.16 | 0.36 | 0.48 |
| Newfoundland | 0.89 | 0.11 | 0.00 | 0.74 | 0.26 | 0.00 | 0.11 | 0.42 | 0.47 |
| Georges Bank | 0.88 | 0.08 | 0.04 | 0.74 | 0.22 | 0.04 | 0.17 | 0.42 | 0.42 |
