# Supplementary material for: The conserved Phe GH5 of importance for hemoglobin intersubunit contact is mutated in gadoid fish
Source: BMC Evol Biol. 2014 Mar 21;14:54. doi: 10.1186/1471-2148-14-54 (PMC3998052; doi:10.1186/1471-2148-14-54)
Supplement: Additional file 6: Table S5 — Observed and expected heterozygosity at three polymorphic positions of Atlantic cod β1 globin in 15 trans-Atlantic populations. All samples conformed to Hardy-Weinberg expectations. [file 1471-2148-14-54-S6.docx]

**Supplementary table S4.**

| **Sample** | **Met55Val** | **Met55Val** | **Lys62Ala** | **Lys62Ala** | **Leu122Met** | **Leu122Met** |
| --- | --- | --- | --- | --- | --- | --- |
| **locations** | ***H*obs** | ***H*exp** | ***H*obs** | ***H*exp** | ***H*obs** | ***H*exp** |
|  |  |  |  |  |  |  |
| Baltic Sea | 0.000 | 0.000 | 0.000 | 0.000 | 0.000 | 0.000 |
| Öresund | 0.604 | 0.500 | 0.604 | 0.495 | 0.000 | 0.000 |
| Kattegat | 0.417 | 0.504 | 0.426 | 0.478 | 0.000 | 0.000 |
| North Sea | 0.538 | 0.501 | 0.590 | 0.505 | 0.000 | 0.000 |
| Faeroe Bank | 0.109 | 0.104 | 0.109 | 0.104 | 0.043 | 0.043 |
| Faeroe Plateau | 0.174 | 0.161 | 0.174 | 0.161 | 0.022 | 0.022 |
| Lofoten Islands | 0.362 | 0.395 | 0.348 | 0.444 | 0.021 | 0.021 |
| Bjørnøya | 0.114 | 0.108 | 0.114 | 0.148 | 0.068 | 0.067 |
| Iceland Coastal | 0.000 | 0.000 | 0.000 | 0.000 | 0.079 | 0.077 |
| Iceland Frontal | 0.053 | 0.052 | 0.079 | 0.077 | 0.237 | 0.212 |
| Nuuk | 0.000 | 0.000 | 0.083 | 0.082 | 0.208 | 0.254 |
| Sisimiut | 0.040 | 0.040 | 0.167 | 0.223 | 0.560 | 0.411 |
| Labrador | 0.080 | 0.078 | 0.360 | 0.301 | 0.360 | 0.458 |
| Newfoundland | 0.105 | 0.102 | 0.263 | 0.235 | 0.421 | 0.444 |
| Georges Bank | 0.083 | 0.156 | 0.217 | 0.264 | 0.417 | 0.479 |
