# Supplementary material for: The conserved Phe GH5 of importance for hemoglobin intersubunit contact is mutated in gadoid fish
Source: BMC Evol Biol. 2014 Mar 21;14:54. doi: 10.1186/1471-2148-14-54 (PMC3998052; doi:10.1186/1471-2148-14-54)
Supplement: Additional file 7: Figure S2 — Multiple sequence alignment of gadiform β1 globins. Nucleotides identical to G. morhua isoform 1 are represented by a dot. Positively selected sites with a Bayesian posterior probability greater than 0.99 are highlighted in gray. Codons 6, 9, 13, 55, 62 and 122 were identified by all evolution models tested. Predicted amino acid sequence of G. morhua isoform 1 together with amino acid substitutions in the positively selected codons are shown in italics above the nucleotide sequence. Genbank accession numbers for the β1 globin nucleotide sequences are as follows: G. morhua (FJ392683, FJ66675, FJ666977, FJ666972, FJ666976, FJ666973), M. merlangus (X98349), B. saida (HO076351, HO075251, HO075774), A. glacialis (DQ125476), M. aeglefinus (F4B3CVS02GS884) and L. lota (F4B3CVS02HILH5). [file 1471-2148-14-54-S7.docx]

***T/Y S/T/K T/Q***

***M V E W T A A E R R H V E A V W S K I D I D V C G P L A L Q R C L I V Y P W T Q***

***G. morhua 1***  ATGGTTGAATGGACAGCTGCTGAGCGGAGGCACGTCGAGGCGGTCTGGAGCAAGATCGACATTGATGTCTGCGGACCACTCGCGTTGCAGAGATGCCTGATTGTGTATCCGTGGACGCAG

***G. morhua 2***  ........................................................................................................................

***G. morhua 3***  ....................C...................................................................................................

***G. morhua 4***  ........................................................................................................................

***G. morhua 5***  ........................................................................................................................

***G. morhua 6***  ........................................................................................................................

***M. merlangus***  ....................C........C...A.....A..A...........................T......G..........................................

***B. saida 1***  ..................A.C..A....CC...A........A.............................................................................

***B. saida 2***  ..................A.C..A....CC...A........A.............................................................................

***B. saida 3***  ..................A.C..A....CC...A........A.............................................................................

***A. glacialis***  ---------------------------------------------...........T..T............................................................

***M. aeglefinus*** ....................C............A.....A..A...........................T......G..........................................

***L. lota***  .......C........A.TAC...A...A.GC.A....CCA.A.........CC...............AT.........A........A......T.A...........C.........

***V/L A/N/Q/R***

***R Y F G S F G D L S T D A A I M G N P K V A K H G V V A L T G L R T A L D H M D***

***G. morhua 1***  CGCTACTTCGGTAGCTTTGGCGACCTGAGCACCGACGCCGCTATTATGGGAAACCCCAAGGTGGCCAAGCACGGCGTCGTGGCCCTGACCGGCCTGAGGACGGCTCTGGACCACATGGAC

***G. morhua 2***  .............................................G...................TGC....................................................

***G. morhua 3***  ........................................................................................................................

***G. morhua 4***  ......................................................................................A.................................

***G. morhua 5***  .............................................G...................TGC....................................................

***G. morhua 6***  ........................................................................................................................

***M. merlangus***  .............................................G.C........T.....................................................T.........

***B. saida 1***  .............................................G.T....................C...................................................

***B. saida 2***  .............................T...............G.T....................C...................................................

***B. saida 3***  .............................................G.T....................C...................................................

***A. glacialis***  ..................................T...............................C.....................................................

***M. aeglefinus*** ....................G........................G.C...................G....................................................

***L. lota***  ....................................AAT......T..A.C.............................C..............A.A..T..........A........

***E I K S T Y A A L S V L H S E K L H V D P D N F R L L C E C L T I V V A G K M G***

***G. morhua 1***  GAAATCAAGTCCACCTACGCTGCCCTGAGCGTGCTGCACTCCGAGAAACTGCACGTCGACCCCGACAACTTCCGACTGCTGTGTGAGTGCCTGACCATTGTCGTCGCCGGGAAGATGGGG

***G. morhua 2***  ........................................................................................................................

***G. morhua 3***  ........................................................................................................................

***G. morhua 4***  ........................................................................................................................

***G. morhua 5***  ........................................................................................................................

***G. morhua 6***  ........................................................................................................................

***M. merlangus***  ..G......G............................................................................C.................................

***B. saida 1***  ..C......G...........A.G..............................................................C.................................

***B. saida 2***  ..C......G...........A.G..............................................................C.................................

***B. saida 3***  ..C......G...........A.G..............................................................C.................................

***A. glacialis***  ......................................................................................................A.................

***M. aeglefinus*** ..G......G............................................................................C.................................

***L. lota***  A.C......AA.G.T...T....T................................................A.............C...........A....................A

***M/C***

***K K L S P E M Q A A W Q K Y L C A V V S A L G R Q Y H***

***G. morhua 1***  AAGAAATTGAGCCCGGAGATGCAGGCTGCGTGGCAGAAGTACCTGTGCGCGGTGGTTTCCGCCCTGGGGAGACAGTACCAC

***G. morhua 2***  .................................................................................

***G. morhua 3***  .................................................................................

***G. morhua 4***  .................................................................................

***G. morhua 5***  ......A..........................................................................

***G. morhua 6***  ......A..........................................................................

***M. merlangus***  ......C....G..C..A..........................C.C..........G.......................

***B. saida 1***  ....C......G..C...............................C..................................

***B. saida 2***  ....C......G..C...............................C..................................

***B. saida 3***  ....C......G..C...G...........................C..................................

***A. glacialis***  ..............C..C...............................................................

***M. aeglefinus*** ......C.......C..A......T...................C.C..........G.......................

***L. lota***  CGC....GC.C............A..C......................T...T..C..T.....C..T..G.........
